# Cytokine RT-qPCR and ddPCR for immunological investigations of the endangered Australian sea lion (*Neophoca cinerea*) and other mammals

**DOI:** 10.7717/peerj.10306

**Published:** 2020-11-13

**Authors:** María-Ignacia Meza Cerda, Rachael Gray, Damien P. Higgins

**Affiliations:** Sydney School of Veterinary Science, Faculty of Science, The University of Sydney, Camperdown, NSW, Australia

**Keywords:** RT-qPCR, ddPCR, Cytokine, Immune response, *Neophoca cinerea*, Interleukin, Cross-reactive, Gene expression, Ecoimmunology, Pinniped

## Abstract

Measurement of cytokine gene expression by reverse transcription quantitative polymerase chain reaction (RT-qPCR) is used widely to assess the immune system of animals and to identify biomarkers of disease, but its application is limited in wildlife species due to a lack of species-specific reagents. The free-ranging endangered Australian sea lion (*Neophoca cinerea*) experiences significant clinical disease and high pup mortality due to intestinal hookworm infection. Developing immunological tools specific to the species will aid in the assessment of drivers of disease and its impact in population demographics. This study describes the development and validation of cross-reactive RT-qPCR assays to measure five important cytokines involved in innate and Th1/Th2 responses (IL-6, TNFα, IFNγ, IL-4 and IL-10) in unstimulated blood samples from a range of different mammalian species including the Australian sea lion. All RT-qPCR assays efficiencies ranged between 87% (*Ovis aries* TNFα) and 111% (*Bos taurus* IL-10) and had strong linearity (*R*^2^). IL-4 and IFNγ gene expression for *N. cinerea* fell below the dynamic range (and therefore quantifiable limits) of RT-qPCR assays but were able to be quantified using the novel droplet digital PCR (ddPCR). This study delivers new immunological tools for eco-immunologists studying cytokine gene expression in wildlife species and is to our knowledge, the first cytokine ddPCR approach to be reported in a pinniped species.

## Introduction

Wildlife species are exposed to a wide range of stressors, often increasing their susceptibility to disease. The endangered Australian sea lion (*Neophoca cinerea*) experiences high pup mortality rates at some colonies in southern Australia, limiting population growth and likely contributing to population decline (Goldsworthy, 2015; Goldsworthy et al., 2009; Marcus, Higgins & Gray, 2014; Shaughnessy et al., 2011). Disease caused by hookworms (*Uncinaria sanguinis*) has been identified as a significant contributor to this trend (Marcus, Higgins & Gray, 2014). Given this endemic pathogen is prevalent at 100% in pups, an understanding of the host response is likely to be informative when evaluating the potential for factors such as anthropogenic pollution, resource or genetic limitations to impact susceptibility to disease. In every species, disease outcomes result, in part, from the interaction between multiple immune cell types and specialised secretory molecules such as acute phase proteins, hormones and cytokines that work together as a network in up-regulated and down-regulated pathways. Cytokines play an essential role in both the initiation and maintenance of the immune response against pathogens, and their variations serve as indicators to assess the immune system of animals and as biomarkers of disease (Fonfara et al., 2008; Fonfara, Siebert & Prange, 2007; Murtaugh et al., 1996; Sitt et al., 2016). Wildlife researchers within the growing discipline of eco-immunology, are using approaches such as the reverse transcription quantitative polymerase chain reaction (RT-qPCR) to measure the expression of cytokine messenger RNA (mRNA) in order to understand the immune system in a wild context (Bowen et al., 2006; Bowen et al., 2012; Brock, Murdock & Martin, 2014; Shoda, Brown & Rice-Ficht, 1998). Although some progress has been made, further research is needed to evaluate the impacts of threats on individual fitness and species resilience (Bowen et al., 2012; Puech et al., 2015; Spitz et al., 2015), so as to guide management decisions aimed toward protecting threatened populations.

Many RT-qPCR protocols have been developed to characterise cytokine gene expression in mice, humans and domestic animals owing to the availability of complete genomic sequences for those species (Boeuf et al., 2005; Murtaugh et al., 1996; Overbergh et al., 1999). However, specific or cross-reactive reagents for threatened wildlife species are still limited (Levin, 2018; Zimmerman et al., 2014). For marine mammals, mRNA expression of cytokines interleukin (IL)-4, IL-10 and tumour necrosis factor (TNF)-α have been examined in cetaceans (Beineke et al., 2007; Chen et al., 2016; Fair et al., 2017; Funke et al., 2002; Inoue et al., 1999; King et al., 1996; Lehnert et al., 2019; St-Laurent, Béliveau & Archambault, 1999). IL-2, IL-10 and, less commonly IL-4, IL-6 and interferon (IFN)-γ have been studied in pinnipeds (Fonfara et al., 2008; Funke et al., 2002; Levin et al., 2014; Shoda, Brown & Rice-Ficht, 1998). These commonly studied cytokines play key roles in orchestrating the balance between critical innate (IL-6, TNFα), T-helper-1 (Th1: IFNγ), T-helper-2 (Th2: IL-4) and T-regulatory (IL-10) immunological pathways. These pathways are interdependent, requiring a finely controlled balance for a productive response, and some overlap in function between resulting immune pathways can occur (Del Prete et al., 1993). In marine mammals, as in other mammals, inflammation is initiated and maintained by signals (e.g. IL-6, TNFα) from sentinel cells of the innate response. Subsequent adaptive responses may focus on Th1 responses (IFNγ) for clearance of intracellular organisms (Fair et al., 2017; Ferrante, Hunter & Wellehan, 2018; Fonfara et al., 2008). Typically, the differentiation of Th2 lymphocytes shifts immunity into a humoral response to combat extracellular pathogens, including parasites (Abo-Aziza et al., 2020; Rostami-Rad, Jafari & Yousofi Darani, 2018). Cytokine IL-4, secreted by Th2 cells, promotes the maturation of B lymphocytes towards plasma cells and immunoglobulin secretion for antibody-mediated responses, long term immunity and repair. This Th2 response is also facilitated by IL-10 which preferentially supresses Th1 responses (Fair et al., 2017; Fonfara et al., 2008).

The majority of cytokine gene expression studies in pinnipeds have been in phocids or involved stimulated peripheral blood mononuclear cells (PMBC) (Das et al., 2008; Fonfara et al., 2008; Levin, 2018). The remote locations and challenging logistics associated with sampling the Australian sea lion and many other wildlife species generally precludes cell separation and culture methods and rather requires the use of whole blood stored in RNA preservative. RT-qPCR is the gold standard for relative gene expression and has become widely used in wildlife (Bowen et al., 2012; Bustin et al., 2005; Funke et al., 2002; Lau et al., 2015; Lehnert et al., 2019) but is limited when applied to samples with low gene transcript concentrations and/or the presence of PCR inhibitors. The more recently developed droplet digital PCR (ddPCR) (Bio-Rad, Hercules, CA, USA) has potential to overcome these limitations by partitioning a normal PCR reaction into thousands of droplets, in which fluorescent dye-based end-point PCRs occur independently, thereby increasing the likelihood of detecting low abundance targets by decreasing the effect of interfering substances and PCR biases (Rački et al., 2014). These features can allow for more precise, reproducible and statistically significant results when working with low levels of nucleic acid and variable amounts of contaminants (Baker et al., 2018; Hindson et al., 2013; Rački et al., 2014; Taylor, Laperriere & Germain, 2017) but the technique has not yet been widely applied in immunology studies of free-ranging animals.

The purpose of this study is to develop and validate RT-qPCR assays to measure, by relative quantification via the delta Ct method, five important cytokines involved in innate and Th1/Th2 responses (IL-6, TNFα, IFNγ, IL-4 and IL-10) in the Australian sea lion. A consensus sequence approach was taken, and the primers’ performance in diverse domestic species was confirmed to illustrate their suitability as candidates for evaluation in future immunological studies of other mammalian wildlife species. Additionally, a subset of these primers was adapted for use in species-specific ddPCR to permit quantification of IL-4 and IFNγ mRNA, which were found to be in low-abundance in blood samples collected from Australian sea lion pups.

## Materials and Methods

### Real-time PCR primer design

Sequences for genes of interest (GOI) IL-4, IL-6, IL-10, IFNγ and TNFα of multiple mammal species were obtained from Genbank (http://www.ncbi.nlm.nih.gov/genbank) and aligned (Table 1).

**Table 1:** Characteristics of consensus primers (IL-4, IL-6, IL-10, IFNγ and TNFα) developed for *Neophoca cinerea, Canis familiaris, Bos taurus*, and *Ovis aries* in the study, and GAPDH (Peters et al., 2004) reference gene primers used in the study.

Gene	GenBank ensemble sequences	Primers (5′->3′) (based on ** marked sequences)	Annealing/extension (°C, s)	Amplicon size (bp)	Conc (nM)	T_m_ (°C)
IL-4	**AF083270.1_*Canis familiaris*	IL-4 F: TCACCTCCCAACTGATTCCAA	60 °C, 30	135	200	82
	EU276069.1_*Bos taurus*					
	NM_001304911.1_*Ailuropoda melanoleuca*					
	KP792599.1_*Neovison vison*					
	XM_027606287.1_*Zalophus californianus*	IL-4 R: ACGAGTCGTTTCTCGCTGTG	60 °C, 30			
	XM_006734683.1_*Leptonychotes weddellii*					
	AB020732.1_*Tursiops truncatus*					
	HM011505.1_*Macropus eugenii*					
IL-6	**L46802.1_*Phoca vitulina*	IL-6 F: CTGCTCCTGGTGATGGCTAC	60 °C, 30	147	200	84.5
	U64794.1_*Equus caballus*					
	AF275796.1_*Canis familiaris*					
	EF368209.1_*Mustela putorius furo*	IL-6 R: TGCAGAGATTTTGCCGAGGA	60 °C, 30			
	EF543744.1_*Ailuropoda melanoleuca*					
	L46803.1_*Orcinus orca*					
IFNγ	**KJ888148.1_*Neovison vison*	IFNγ F: GTGAATGATCTGCAGGTCCA	60 °C, 30	101	200	80
	NM_213948.1_*Sus scrofa*					
	NM_001003174.1_*Canis familiaris*					
	XM_026500086.1_*Ursus arctos*					
	XM_027593881.1_*Zalophus californianus*	IFNγ R: TGACTCCTTTTCCGCTTCCT	60 °C, 30			
	XM_025883084.1_*Callorhinus ursinus*					
	XM_006740002.1_*Leptonychotes weddellii*					
	DQ118388.1_*Phoca vitulina*					
IL-10	**NM_001003077.1_*Canis familiaris*	IL-10 F: CTTTAAGAGTTACCTGGGTTGCC	60 °C, 30	97	200	83.5
	DQ890062.1_*Macaca mulatta*					
	NM_001082490.1_*Equus caballus*					
	XM_002919274.3_*Ailuropoda melanoleuca*					
	XM_027613593.1_*Zalophus californianus*	IL-10 R: GATGTCTGGGTCGTGGTTCTC	60 °C, 30			
	XM_004417581.2_*Odobenus rosmarus*					
	L46802.1_*Phoca vitulina*					
	AF026277.1_*Trichosurus vulpecula*					
TNFα	**XM_027099307_*Lagenorhynchus obliquidens*	TNFα F: GAGCACTGAAAGCATGATCCG	60 °C, 30	123	200	87
	D86587.1_*Capra hircus*					
	NM_001003244.4_*Canis familiaris*					
	EF368211.1_*Mustela putorius furo*					
	XM_002930032.3_*Ailuropoda melanoleuca*	TNFα R: GCGACCAGGAAGAAGGAGAA	60 °C, 30			
	XM_027603858.1_*Zalophus californianus*					
	XM_025862490.1_*Callorhinus ursinus*					
	XM_006738478.1_*Leptonychotes weddellii*					
GAPDH	Peters et al. (2004)	GAPDH F: TCAACGGATTTGGCCGTATTGG	60 °C, 30	90	400	83.5
		GAPDH R: TGAAGGGGTCATTGATGGCG				

**Notes:**

T_m_, melting temperature.

** Marked sequences: IL-4 AF083270.1_*Canis familiaris*; IL-6 L46802.1_*Phoca vitulina*; IFNγ KJ888148.1_*Neovison vison*; IL-10 NM_001003077.1_*Canis familiaris*; TNFα XM_027099307_*Lagenorhynchus obliquidens*.

Primers for qPCR (Macrogen, Seoul, South Korea) (Table 1) were designed within conserved regions using NCBI Primer-BLAST (Ye et al., 2012) and recommended parameters for designing SYBR^®^ Green primers (Thornton & Basu, 2011). Secondary structures and specificity against non-specific sequences for each primer set were assessed in silico using BeaconDesigner™ (http://www.premierbiosoft.com/qOligo/Oligo.jsp?PID=1) and NCBI Primer-BLAST (Ye et al., 2012), respectively. Glyceraldehyde 3-phosphate dehydrogenase (GAPDH) was selected as a reference gene for this study given its stability in a variety of sample types and its validation in many species including domestic animals, marsupials and marine mammals (Beineke et al., 2004; Fonfara et al., 2008; Maher et al., 2014; Puech et al., 2015; Sharp et al., 2006). Primers for GAPDH (Macrogen, Seoul, South Korea) were selected from a previous publication that used Genbank sequences for *Canis familiaris* (Peters et al., 2004) and its performance was optimised to the study conditions.

### Blood collection, RNA extraction and reverse transcription

Blood samples (0.5 mL) from domestic dog (*C. familiaris, n* = 4), cattle (*Bos Taurus*, *n* = 3) and sheep (*Ovis aries*, *n* = 3) were collected from brachial, tail and jugular veins, respectively into EDTA tubes (Sarstedt, Nümbrecht, Germany) and then centrifuged at 5,000×*g* for up to 3 min. Plasma was removed using a sterile disposable pipette, and the remaining red blood cells and buffy coat were resuspended in 1,300 µl RNAlater^™^ (Applied Biosystems, Carlsbad, CA, USA) and then transferred into cryovials to approximate field storage conditions. Samples were initially stored at 4 °C for 2–4 days and then at −20 °C until RNA extraction was performed within 12 months of blood collection. RNA extractions from the same species were combined and used as pooled samples for further applications.

In 2010, four blood samples (0.5 mL) collected from the brachial vein from *N. cinerea* pups for a previous study (Marcus, Higgins & Gray, 2014) (Government of South Australia Department of Environment, Water and Natural Resources; Wildlife Ethics Committee approvals 3–2008 and 3–2011 and Scientific Research Permits A25088/4–5) were immediately transferred into EDTA tubes (Sarstedt, Nümbrecht, Germany) and processed as described above. Samples were initially stored at 4 °C for 2–4 days and then at −20 °C until RNA extraction was performed in 2018. RNA extractions were combined and used as pooled samples for further applications.

For RNA extractions, samples in RNAlater^™^ were thawed at room temperature, centrifuged at 16,000×*g* for 1 min, and the supernatant of RNAlater^™^ discarded from the cell pellet. Total RNA extraction was performed on the cell pellet using the RiboPure^™^-Blood Kit (Ambion, Carlsbad, CA, USA) according to the manufacturer’s instructions, such that the equivalent of 200 µl of whole blood was used per extraction. The RNA concentration and purity were assessed (*A*_260_/*A*_280_) using a NanoDrop 1000, Thermo Scientific^™^ (Waltham, MA, USA) and RNA stored as multiple aliquots at −80 °C for subsequent use.

For analysis, aliquots of RNA were thawed on ice, and two sequential DNase treatments were performed using the RNase-free DNase I (provided in the RNA extraction kit) to eliminate genomic DNA (gDNA). Reverse transcription was performed on 50–100 ng of RNA template in a 20 µl reaction with the RevertAid First Strand cDNA Synthesis Kit (Thermo Fisher^™^, Carlsbad, CA, USA), with a combination (50:50) of random hexamer and oligo(dT)_18_ primers to improve the sensitivity of cDNA synthesis (Ferrante, Hunter & Wellehan, 2018; Gallup, 2011). cDNA was stored as multiple aliquots at −20 °C for subsequent use.

### Real-time PCR optimisation and validation

Optimisation and validation parameters were achieved following recommendations for qPCR assays from Bustin et al. (2009) (MIQE guidelines). All qPCR assays were performed using SYBR Green (SsoAdvanced^™^ Universal SYBR^®^ Green Supermix, BioRad) on a CFX96 Real-Time cycler (Biorad, Hercules, CA, USA). Cycling conditions were optimised by annealing temperature (*T*_a_) gradient and evaluation of three primer concentrations (100, 200 and 300 nM). Optimal parameters were determined based on those that yielded a single, sharp peak in the melt curve analysis with the lowest quantification cycle (Ct) for each primer pair (Table 1).

Amplifications were performed in white 8-strip PCR tubes (BioRad, California, USA), following manufacturer’s instructions for the SYBR Green Supermix in a 20 µl reaction with each primer pair at optimised concentration and 4 µl of cDNA template. In addition, “no-reverse transcription” controls (NRT), and “no template” controls (NTC) of RNase-DNase free water, were included in each run to check for gDNA contamination and the formation of primer-dimers, respectively. Under identical qPCR cycling conditions, reactions were validated across cDNA templates from *N. cinerea, C. familiaris, B. taurus* and *O. aries*. Amplification conditions were 95 °C for 1 min (1 cycle); 95 °C for 10 s and 60 °C for 20 s (40 cycles). After each cycling protocol, a melt curve analysis was generated by heating from 65 °C to 95 °C with 0.5 °C increments for 5 s to confirm the absence of non-specific products or primer dimers and define melting temperatures (*T*_m_) for each amplicon. The size of each qPCR product was confirmed by 2% agarose gel electrophoresis and identity of the amplicon was further confirmed by DNA sequence analysis (Macrogen, Seoul, South Korea) and comparison with nucleotide sequences of terrestrial and marine mammals using the NCBI BLAST programme (Altschul et al., 1990).

Confirmed qPCR products were removed from the plates and diluted for further use as a template in standard curves. Amplification efficiencies for each gene of interest (GOI) and the reference gene (GAPDH) were determined for each species through a standard curve using serial dilutions of qPCR product with a minimum of five standards with the dilution extending at least to that producing a Ct of 34. The efficiency (*E*) and linearity (*R*^2^) of each primer pair were calculated on these curves using the Bio-Rad CFX Maestro software 1.1 (BioRad, Hercules, CA, USA) with automatic threshold settings. Linear regression of the qPCR standard curves was recalculated with Microsoft Excel (Microsoft, Redmond, WA, USA; 2016). Primer pairs with efficiencies of 100% ± 10% and *R*^2^ value > 0.96 were considered optimised for qPCR (IL-4, IL-6, IL-10, IFNγ and TNFα) following MIQE recommended ranges. The limit of quantification (LoQ) was based on the linear operating range of each assay (Berdal & Holst-Jensen, 2001). Assays that showed linear and efficient amplification but that produced Ct values above the dynamic range of qPCR (Ct alues >35) when applied to *N. cinerea* pup blood samples, were selected for subsequent development of novel ddPCR. As the assays were developed for use in relative expression studies using the delta Ct method, absolute quantification and limits of detection (LOD) were not derived using quantified standards. Quantified standards were, however used for direct comparison of sensitivity of ddPCR vs qPCR in IL-4 and IFNγ assays.

### ddPCR assays for *Neophoca cinerea*

qPCR products obtained from the amplification of *N. cinerea* samples with the IL-4 and IFNγ qPCR primers formerly described in this study were sequenced and aligned using the CLC Main Workbench 6.9.1 (Qiagen, Redwood City, CA, USA). NCBI Primer-BLAST programme (Ye et al., 2012) was used to design *N. cinerea* primer-probe pairs for ddPCR following recommended parameters from the Droplet Digital^™^ PCR Applications Guide (www.bio-rad.com). The primers and probes sequences (Macrogen, Seoul, South Korea) are listed in Table 2. The in silico tool ‘PCR Primer Stats’ (http://www.bioinformatics.org/sms2/pcr_primer_stats.html) was used to evaluate primer-probe pairs melting temperature, GC content and secondary structures (Stothard, 2000). Probes were labelled with FAM (F) fluorophore and quenched with non-fluorescent black hole quenchers number 1 (BHQ-1).

**Table 2:** Characteristics of cytokine primers developed in the study for ddPCR for IL-4 and IFNγ in *N. cinerea*.

Gene name	Primer sequences (5′->3′)	Probe fluorophore	Annealing/Extension (°C, s)	Optimal primer/probe concentration (nM)	Amplicon size (Bp)
IL-4	F: TCACCTCCCAACTGATTCCAA		60, 20	400	132
	R: ACGAGTCGTTTCTCGCTGT		60, 20	400	
	**P: GCACTCACCAGCACCTTTGTCCA**	FAM	60, 20	100	
IFNy	F: AGCTGATTCGAATTCCCGTGA		58, 20	400	95
	R: TCTGACTCCTTTTCCGCTTCC		58, 20	400	
	**P: TGCAGGTCCAGCGCAAAGCGATA**	FAM	58, 20	100	

**Figure 1:**
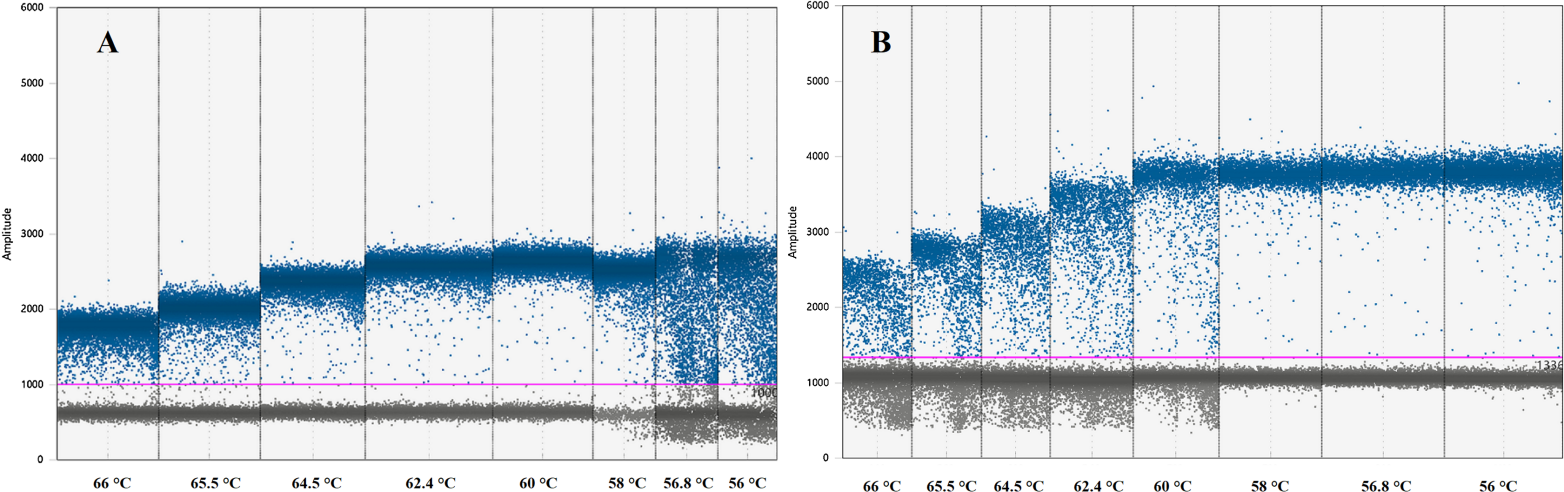
Graph displaying fluorescence amplitude plotted against the annealing temperature gradient for digital droplet PCR. Blue dots above the pink line (threshold) represents positive PCR amplification droplets for (A) IL-4 and (B) IFNγ. Grey dots represent negative droplets. Each column represents one of eight ddPCR reactions across an annealing temperature gradient. The optimal annealing temperature giving the largest difference in fluorescence between negative and positive droplets was 60 °C for (A) IL-4 and 58 °C for (B) IFNγ. Both assays can work simultaneously at 59 °C.

Instruments, reagents and consumables for the ddPCR workflow were supplied by Bio-Rad (Bio-Rad, Hercules, CA, USA). Optimal ddPCR annealing temperatures for IL-4 and IFNγ assays were defined by performing a temperature gradient in the annealing/extension step of the thermal cycling protocol as suggested by ddPCR guidelines (Huggett et al., 2013). All ddPCR optimisation assays were performed using the ddPCR^™^ Supermix for Probes (no dUTP) master mix in a C1000 Touch^™^ Thermal Cycler. The fluorescence difference between NTC and positive samples within a single run was used to set a threshold between negative and positive droplets. Positive droplets show increased fluorescent amplitude when compared to the negative droplets and contain at least one copy of the target per sample. The optimal annealing temperature for these assays was defined as the one giving the largest difference in fluorescence between negative and positive droplets (Table 2; Fig. 1) (Huggett et al., 2013).

ddPCR master mix reactions included 10 µl of the Supermix, one µl of each primer and probe, six µl of cDNA from pooled *N. cinerea* samples and RNase-DNase free water to complete a 20 µl total volume reaction (Table 2). PCR mix and Droplet Generation oil for Probes were added to corresponding wells in the Droplet Generator DG8^™^ Cartridge and covered with DG8^™^ Gaskets following the manufacturer’s instructions. Droplets were generated by using the QX100^™^ Droplet Generator, gently transferred to a clean 96-well PCR plate and sealed using a PCR Plate Sealer. Amplification was performed using the following cycling conditions: an initial enzyme activation period of 10 min at 95 °C, followed by 40 cycles consisting of denaturation at 95 °C for 30 s and annealing/extension step of 59 °C for 1 min, and followed by an enzyme deactivation period of 10 min at 95 °C and a 4 °C indefinite hold. The overall ramp rate was set at 2 °C s^−1^. Droplets were read as positive or negative with a QX200 Droplet Reader, and further analysis was performed using the Quanta-Soft Analysis Pro^™^ software (Bio-Rad). The software, based on fluorescence amplitude, establishes a threshold between positive and negative droplets. Positive droplets are converted to copy numbers in the PCR mix based on Poisson algorithms (Droplet Digital^™^ PCR Applications Guide).

To assess linearity (*R*^2^), efficiency (*E*) and to compare the limits of detection (LoD) and quantification (LoQ) of qPCR and ddPCR, IL-4 and IFNγ consensus sequences were selected to synthesise double-stranded DNA standards (gBlocks^®^ gene fragments; Integrated DNA Technologies, Singapore). Each DNA standard was resuspended in Tris EDTA buffer (The Bosch Institute, Faculty of Medicine, The University of Sydney) to reach a final concentration of 10 ng µl^−1^ according to the manufacturer’s instructions with subsequent storage at −20 °C. For qPCR, a calibration curve (regression line) was performed with 10-fold serial dilutions of the standard ranging from 10^6^ to one target copies µl^−1^ and six technical replicates for each dilution. For ddPCR, 5-fold serial dilutions ranged from 200 to 0 target copies µl^−1^ with three replicates for each dilution. LoD and LoQ were calculated with Microsoft Excel (2016) as 3.3 and 10 times, respectively, the standard deviation of the y-intercept of the regression line, divided by the slope of the corresponding calibration curve (FDA, 1996; Mohamad, 2018).

## Results

### Real-time qPCR design and validation

The primer pairs designed in this study measured cytokine gene expression in mammalian species in separate evolutionary clades, representing terrestrial (domestic dog, sheep and cattle) and the Australian sea lion. mRNA sequences from qPCR products showed high homology to available mammalian sequence data, with values ranging from 86 to 100% (Table 3).

**Table 3:** Nucleotide identity of qPCR products for IL-4, IL-6, IFNγ, IL-10 and TNFα represented by the best match in BLASTn search (http://www.ncbi.nlm.nih.gov/BLAST/) in the four target species.

Target gene	qPCR product	% Best match	Accession Number
IL-4	*Neophoca cinerea*	98	*Eumetopias jubatus* XM_028103636.1
	*Canis familiaris*	95	*Canis familiaris* EF095771.1
IL-6	*Neophoca cinerea*	100	*Zalophus californianus* XM_027574842.1
	*Canis familiaris*	88	*Canis familiaris* AF349534.1
IFNγ	*Neophoca cinerea*	98	*Enhydra lutris* XM_022495107.1
	*Canis familiaris*	97	*Canis lupus* XM_025476664.1
IL-10	*Neophoca cinerea*	100	*Eumetopias jubatus* XM_028120121.1
	*Canis familiaris*	95	*Canis familiaris* EU426968.1
	*Bos taurus*	95	*Bos indicus* KX013148.1
	*Ovis aries*	97	*Equus caballus* XM_023640225.1
TNFα	*Neophoca cinerea*	90	*Eumetopias jubatus* XM_028120686.1
	*Canis familiaris*	87	*Vulpes vulpes* KM892854.1
	*Bos taurus*	95	*Bos taurus* NM_173966.3
	*Ovis aries*	86	*Ovis aries* EF446377.1

The integrity of isolated RNA was demonstrated in all blood samples from every species by the amplification of GAPDH mRNA (Ct 25 ± 3.1, mean ± SD). Optimal qPCR parameters allowed amplification of IL-4 (Australian sea lion and dog), IL-6 (Australian sea lion and dog), IL-10 (all four targeted species), TNFα (all four targeted species), IFNγ (Australian sea lion and dog) and GAPDH (all four targeted species), using the same cycling protocol, with a combined annealing and extension step at 60 °C. The defined optimal parameters for the assays are shown in Table 1. In addition, the presence of a single specific product was confirmed by melt curve analysis (Figs. S1–S4), agarose gel electrophoresis and sequencing. The absence of non-specific products and primer-dimers was confirmed (Figs. S1–S4). No amplification was detected in NRT controls and NTC. Two consecutive DNAse treatments were required to eliminate evidence of gDNA in NRT controls. All assay efficiencies ranged between 87% (*Ovis aries* TNFα) and 111% (*Bos taurus* IL-10) and had strong linearity (*R*^2^) (Table 4). Although sample sizes were too small for comparison, the low expression of IL-4 and IFNγ in *N. cinerea* was consistent with levels in canine samples that we assessed (Ct 32 ± 2.5, *n* = 4).

**Table 4:** qPCR: efficiency (*E*%) and linearity (*R*^2^) for IL-4, IL-6, IFNγ, IL-10, TNFα and GAPDH in their respective targeted species.

Target gene	Species	Slope	*R*^2^	*E* (%)
IL-4	*Neophoca cinerea*	−3.267	0.987	102
	*Canis familiaris*	−3.244	0.990	103
IL-6	*Neophoca cinerea*	−3.293	0.985	101
	*Canis familiaris*	−3.351	0.999	99
IFNγ	*Neophoca cinerea*	−3.229	0.999	104
	*Canis familiaris*	−3.225	0.996	103
IL-10	*Neophoca cinerea*	−3.132	0.996	95
	*Canis familiaris*	−3.319	0.998	100
	*Bos taurus*	−3.083	0.994	111
	*Ovis aries*	−3.167	0.996	107
TNFα	*Neophoca cinerea*	−3.374	0.989	98
	*Canis familiaris*	−3.271	0.936	102
	*Bos taurus*	−3.624	0.998	89
	*Ovis aries*	−3.684	0.999	87
GAPDH	*Neophoca cinerea*	−3.385	0.992	106
	*Canis familiaris*	−3.581	0.998	87
	*Bos taurus*	−3.288	0.979	101
	*Ovis aries*	−3.371	0.998	98

### ddPCR assays for *Neophoca cinerea*

The ddPCR primers and probe assays designed in this study effectively amplified *N. cinerea* blood mRNA. The forward-reverse and probe sequences of each assay are listed in Table 2. Gene sequence data obtained for both primer-probe pairs was BLASTn compared against similar mammalian species genes, and the results show 98% homology with marine mammals (Table 5). No amplification was detected in NTC.

**Table 5:** BLASTn results of identity for *Neophoca cinerea* qPCR amplified genes represented by the best match with pinnipeds’ sequences in BLASTn search (http://www.ncbi.nlm.nih.gov/BLAST/).

Target gene	% Best match	Accession Number
IL-4	98	*Eumetopias jubatus* XM_028103636.1
IFNγ	98	*Phoca vitulina* XM_032420714.1

**Figure 2:**
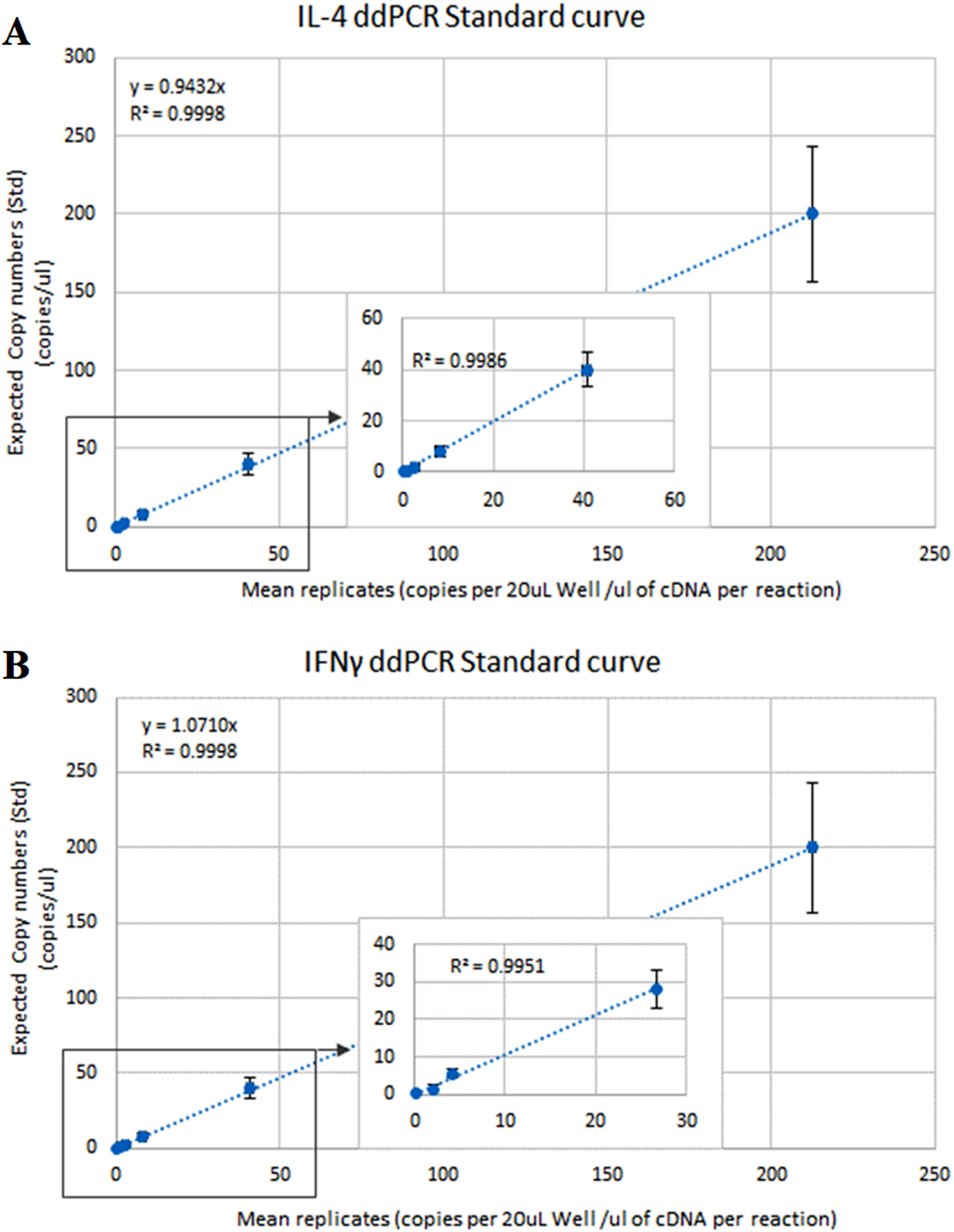
ddPCR calibration curves (regression lines) for the calculation of the Limit of Detection (LoD) and Limit of Quantification (LoQ) for IL-4 and IFNγ assays in *Neophoca cinerea*. The *x*-axis represents the average (triplicates) copies of (A) IL-4 and (B) IFNγ per 20 μL reaction to the quantity of cDNA used in the PCR reaction (Quanta-Soft Analysis ProTM™ software). The *y*-axis represents 5-fold series dilutions of DNA standards. Inset graphs show linearity of the assays in the more diluted standards.

The optimal ddPCR annealing/extension temperature for IL-4 and IFNγ defined by a temperature gradient was 60°C and 58°C, respectively (Fig. 1). The defined optimal parameters for the assays are shown in Table 2. LoD and LoQ were confirmed to be lower in ddPCR than in qPCR (Table 6; Fig. 2). Quantification of IFNγ and IL-4 in the pooled *N. cinerea* samples indicated 69 and 13 copies per reaction, respectively (Table 6).

**Table 6:** qPCR and ddPCR validation parameters for IL-4 and IFNγ primer-probe pairs in *Neophoca cinerea*.

qPCR					ddPCR		
Target gene	*E* (%)	*R*^2^	LoD	LoQ	Copies in pooled samples	LoD	LoQ
			**(Copy numbers)**			**(Copy numbers)**	
IL-4	101	0.996	6	199	13	4.19	12.68
IFNγ	101	0.997	5	119	69	3.48	10.54

**Note:**

qPCR efficiency (*E*%), linearity (*R*^2^), Limit of Detection (LoD) and Limit of Quantification (LoQ). ddPCR Quanta-Soft Analysis ProTM™ software copy number per reaction for *Neophoca cinerea* pooled samples and LoD and LoQ of the assays.

## Discussion

Cytokine mRNA qPCR has become a broadly used and affordable tool to establish cytokine expression profiles in animals but has been limited by the lack of species-specific reagents (Ferrante, Hunter & Wellehan, 2018; Funke et al., 2002; Maher et al., 2014; Maissen-Villiger et al., 2016; Puech et al., 2015). In this study, optimisation and validation of SYBR green RT-qPCR assays, cross-reactive to diverse mammalian species, was performed to allow relative quantification of mRNA of five important cytokines (i.e. IL-4, IL-6, IL-10, IFNγ and TNFα). Primer sets for IL-4, IL-6 and IFNγ cross-reacted in dog and Australian sea lion samples, whereas those for IL-10 and TNFα also amplified across sheep and cattle. The broad cross-reactivity suggests that these primer sets are likely to cross react across several other mammalian species.

Studies of basal cytokine levels in marine mammals are forthcoming but still limited (Beineke et al., 2007; Beineke et al., 2004; Ferrante, Hunter & Wellehan, 2018; Hofstetter et al., 2017) and, consistent with our study, others have encountered challenges with sensitivity. Sitt et al. (2016) determined that IL-4 transcripts were typically absent in killer whales (*Orcinus orca*) and levels of IL-4 in domestic pig (*Sus scrofa*) remained low even after lymphocyte stimulation (Murtaugh et al., 1996). In the present study, qPCR of mRNA from whole blood was quantifiable for IL-6, IL-10, TNFα and GAPDH but despite optimal specificity and efficiency, IL-4 and IFNγ Ct values for samples from *N. cinerea* pups were outside the limits of quantification (>35). Separation and stimulation of peripheral blood mononuclear cells (PMBCs) is often performed for the quantification of cytokine gene expression (Beineke et al., 2004; King et al., 1993; Levin et al., 2014) and yields much greater concentrations of target mRNA; but these methods are often not feasible under field conditions and may not represent natural expression as closely as cytokine levels in non-stimulated whole blood samples. The novel ddPCR assays overcame this limitation for samples collected from Australian sea lion pups. This method represents a very sensitive technique capable of assessing low abundant targets, confirmed by the lower LoD and LoQ than qPCR. Thus, the technique shows a lot of promise for cytokine gene expression studies involving low abundant targets or samples with carryover of inhibitors from RNA extraction or cDNA synthesis methods, as can occur when field samples associated with wildlife studies cannot be collected and stored under ideal conditions (Rački et al., 2014; Taylor, Laperriere & Germain, 2017).

It was not possible to ensure that the primer pairs developed in this study did not span exon-exon junctions. It is therefore recommended that a thorough removal of gDNA is performed when following the methods presented here. Similar to a previous study Schwochow et al. (2012), two DNase treatments were needed to ensure the removal of contaminating gDNA.

The set of primers developed in this study have potential applications to immunological studies across multiple species, including wildlife, expanding the toolkit for researchers in the future to identify immunological markers of innate, T-helper-1 and T-helper-2 pathways in mammals. To our knowledge, the ddPCR assay developed for Australian sea lions is the first one to be reported in a pinniped species and is presented as an alternative for samples that contain a low concentration of target or those that could be affected by inhibitors. However, further investigation is necessary to explore the full potential of this approach.

## Conclusion

In summary, SYBR Green RT-qPCR assays were developed to quantify cytokine gene expression across diverse mammalian species. The diversity of species strongly suggests that the assays have potential for application beyond the Australian sea lion to many other threatened wildlife species. The sensitivity of methods described here indicates that most are of use in mRNA extracts from whole blood, increasing their utility for analysis of field samples, where immediate sample processing is limited. Conveniently, they can also be applied under the same optimised cycling conditions in their respective targeted species.

The novel ddPCR methods described here enabled detection of low expressed genes, like IL-4 and IFNγ in *N. cinerea* pups, providing comparative advantages when working with unstimulated tissues and limiting sample volumes as in the case of fieldwork-based wildlife studies.

## Supplemental Information

10.7717/peerj.10306/supp-1 Supplemental Information 1 Replicated specificity of qPCR primers for IL-4, IL-6, IFNγ, IL-10, TNFα and GAPDH in *Neophoca cinerea*.. Melting curves of dilutions from standard curves for each target. In the x-axis, single visible peaks represent the melting temperature (Tm) of the double-stranded DNA complexes. The y-axis represents the Relative Fluorescence unit (RFU) (-d(RFU)/dT).

10.7717/peerj.10306/supp-2 Supplemental Information 2 Replicated specificity of qPCR primers for IL-4, IL-6, IFNγ, IL-10, TNFα and GAPDH in *Canis familiaris*. Melting curves of dilutions from standard curves for each target. In the x-axis, single visible peaks represent the melting temperature (Tm) of the double-stranded DNA complexes. The y-axis represents the Relative Fluorescence unit (RFU) (-d(RFU)/dT).

10.7717/peerj.10306/supp-3 Supplemental Information 3 Replicated specificity of qPCR primers for IL-10, TNFα and GAPDH in *Bos taurus*. Melting curves of dilutions from standard curves for each target. In the x-axis, single visible peaks represent the melting temperature (Tm) of the double-stranded DNA complexes. The y-axis represents the Relative Fluorescence unit (RFU) (-d(RFU)/dT).

10.7717/peerj.10306/supp-4 Supplemental Information 4 Replicated specificity of qPCR primers for IL-10, TNFα and GAPDH in *Ovis aries*. Melting curves of dilutions from standard curves for each target. In the x-axis, single visible peaks represent the melting temperature (Tm) of the double-stranded DNA complexes. The y-axis represents the Relative Fluorescence unit (RFU) (-d(RFU)/dT).

10.7717/peerj.10306/supp-5 Supplemental Information 5 Limit of detection and limit of quantification for qPCR primers IL-4 and IFNy. Excel spreadsheets with the standard curves and calculations for Limit of detection and limit of quantification for IL-4 and IFNy primer sets described in the manuscript.

10.7717/peerj.10306/supp-6 Supplemental Information 6 Limit of detection and limit of quantification for ddPCR primers IL-4 and IFNy. Excel spreadsheets with the standard curves and calculations for Limit of detection and limit of quantification for IL-4 and IFNy primer sets described in the manuscript.

10.7717/peerj.10306/supp-7 Supplemental Information 7 Australian sea lion (*Neophoca cinerea*) raw data used to calculate qPCR efficiencies and R^2^ for each of the assays.

10.7717/peerj.10306/supp-8 Supplemental Information 8 Dog (*Canis familiaris*) raw data used to calculate qPCR efficiencies and R^2^ for each of the assays.

10.7717/peerj.10306/supp-9 Supplemental Information 9 Cattle (*Bos taurus*) raw data used to calculate qPCR efficiencies and R^2^ for each of the assays.

10.7717/peerj.10306/supp-10 Supplemental Information 10 Sheep (*Ovis aries*) raw data used to calculate qPCR efficiencies and R^2^ for each of the assays.

10.7717/peerj.10306/supp-11 Supplemental Information 11 qPCR Sequences *Neophoca cinerea*. qPCR *Neophoca cinerea* sequence analysis results (Macrogen, Seoul, South Korea).

## Additional Information and Declarations

### Field Study Permissions

The following information was supplied relating to field study approvals (i.e., approving body and any reference numbers): Fieldwork was approved by the Government of South Australia Department of Environment, Water and Natural Resources [Scientific Research Permits (A25088/4–5)].

### Data Availability

The following information was supplied regarding data availability: Raw data are available in the Supplemental Files.
