# Supplementary figures and images for: Cytokine RT-qPCR and ddPCR for immunological investigations of the endangered Australian sea lion (Neophoca cinerea) and other mammals
Source: PeerJ. 2020 Nov 13;8:e10306. doi: 10.7717/peerj.10306 (PMC7668205; doi:10.7717/peerj.10306)

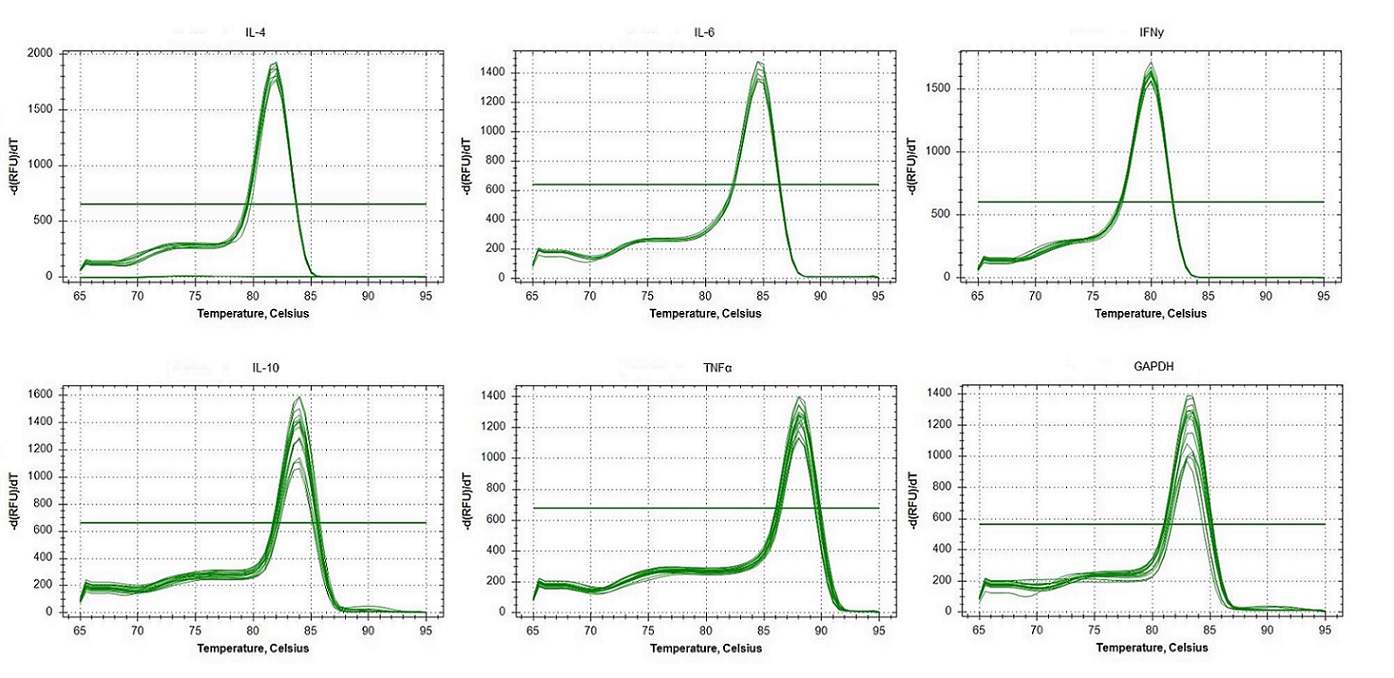

Supplement: Supplemental Information 1 — Melting curves of dilutions from standard curves for each target. In the x-axis, single visible peaks represent the melting temperature (Tm) of the double-stranded DNA complexes. The y-axis represents the Relative Fluorescence unit (RFU) (-d(RFU)/dT). [file peerj-08-10306-s001.jpg]

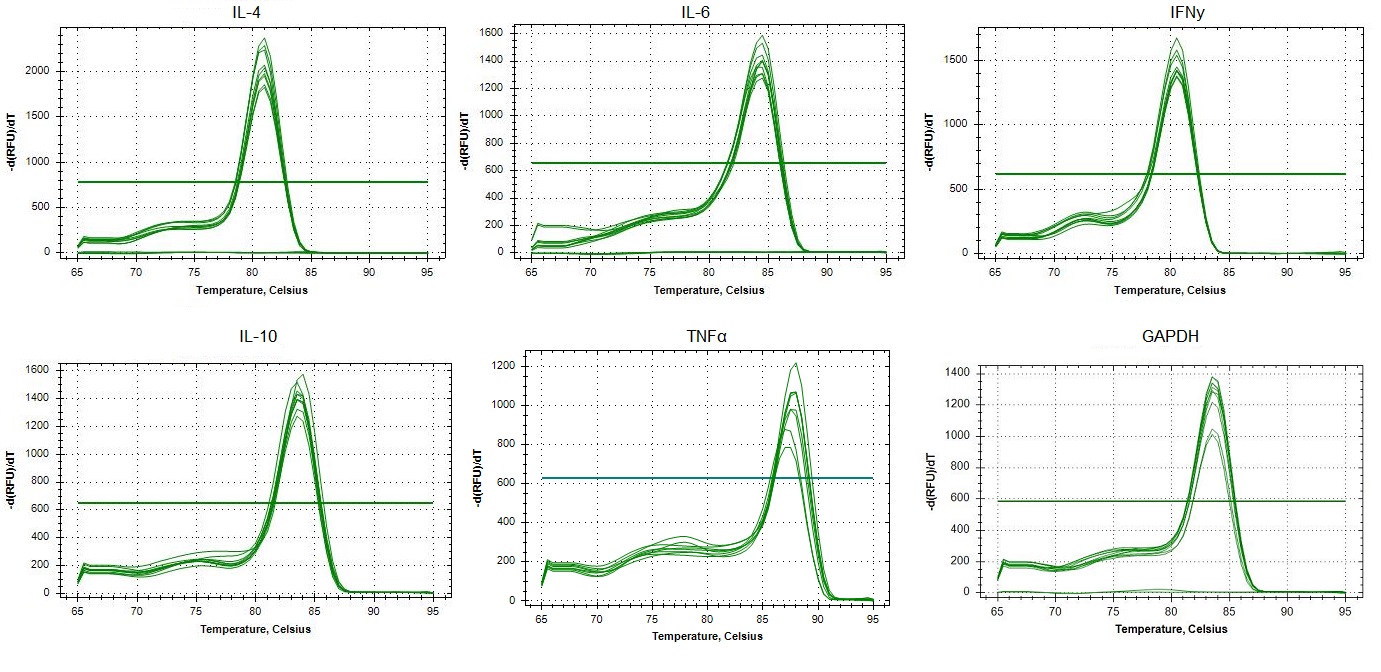

Supplement: Supplemental Information 2 — Melting curves of dilutions from standard curves for each target. In the x-axis, single visible peaks represent the melting temperature (Tm) of the double-stranded DNA complexes. The y-axis represents the Relative Fluorescence unit (RFU) (-d(RFU)/dT). [file peerj-08-10306-s002.jpg]

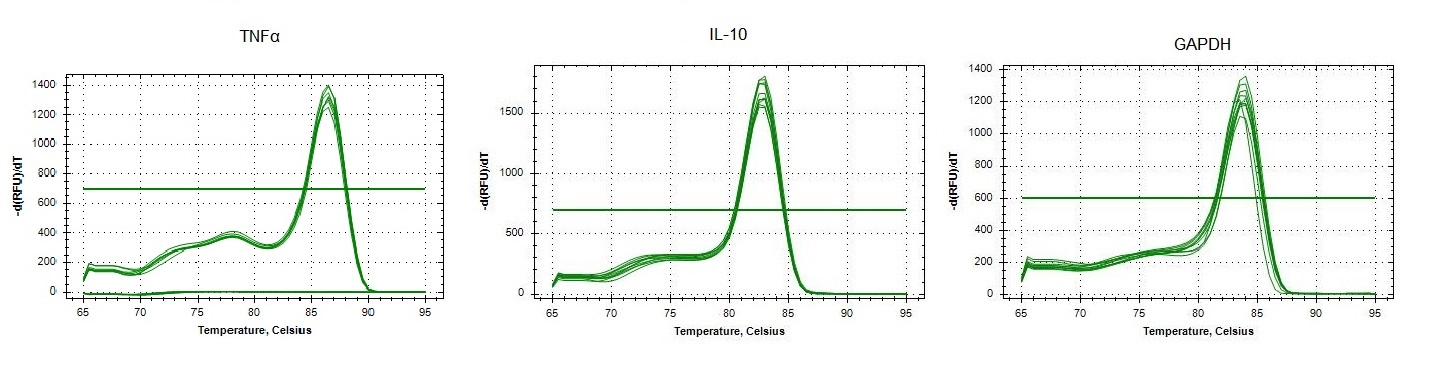

Supplement: Supplemental Information 3 — Melting curves of dilutions from standard curves for each target. In the x-axis, single visible peaks represent the melting temperature (Tm) of the double-stranded DNA complexes. The y-axis represents the Relative Fluorescence unit (RFU) (-d(RFU)/dT). [file peerj-08-10306-s003.jpg]

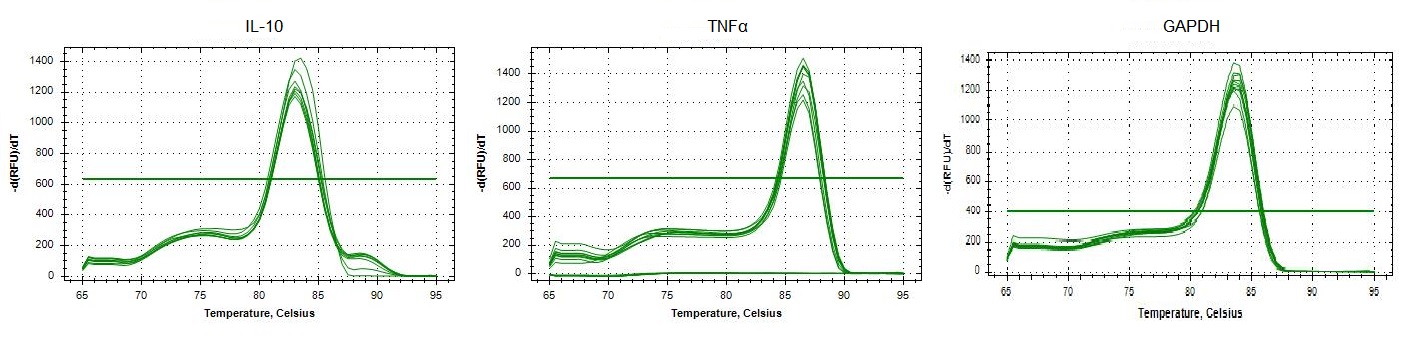

Supplement: Supplemental Information 4 — Melting curves of dilutions from standard curves for each target. In the x-axis, single visible peaks represent the melting temperature (Tm) of the double-stranded DNA complexes. The y-axis represents the Relative Fluorescence unit (RFU) (-d(RFU)/dT). [file peerj-08-10306-s004.jpg]
